# Utilizing elementary mode analysis, pathway thermodynamics, and a genetic algorithm for metabolic flux determination and optimal metabolic network design

**DOI:** 10.1186/1752-0509-4-49

**Published:** 2010-04-23

**Authors:** Brett A Boghigian, Hai Shi, Kyongbum Lee, Blaine A Pfeifer

**Affiliations:** 1 Tufts University, Department of Chemical & Biological Engineering, Science & Technology Center, 4 Colby Street, Medford, MA 02155, USA

## Abstract

**Background:**

Microbial hosts offer a number of unique advantages when used as production systems for both native and heterologous small-molecules. These advantages include high selectivity and benign environmental impact; however, a principal drawback is low yield and/or productivity, which limits economic viability. Therefore a major challenge in developing a microbial production system is to maximize formation of a specific product while sustaining cell growth. Tools to rationally reconfigure microbial metabolism for these potentially conflicting objectives remain limited. Exhaustively exploring combinations of genetic modifications is both experimentally and computationally inefficient, and can become intractable when multiple gene deletions or insertions need to be considered. Alternatively, the search for desirable gene modifications may be solved heuristically as an evolutionary optimization problem. In this study, we combine a genetic algorithm and elementary mode analysis to develop an optimization framework for evolving metabolic networks with energetically favorable pathways for production of both biomass and a compound of interest.

**Results:**

Utilization of thermodynamically-weighted elementary modes for flux reconstruction of *E. coli *central metabolism revealed two clusters of EMs with respect to their Δ*G*_*p*_°. For proof of principle testing, the algorithm was applied to ethanol and lycopene production in *E. coli*. The algorithm was used to optimize product formation, biomass formation, and product and biomass formation simultaneously. Predicted knockouts often matched those that have previously been implemented experimentally for improved product formation. The performance of a multi-objective genetic algorithm showed that it is better to couple the two objectives in a single objective genetic algorithm.

**Conclusion:**

A computationally tractable framework is presented for the redesign of metabolic networks for maximal product formation combining elementary mode analysis (a form of convex analysis), pathway thermodynamics, and a genetic algorithm to optimize the production of two industrially-relevant products, ethanol and lycopene, from *E. coli*. The designed algorithm can be applied to any small-scale model of cellular metabolism theoretically utilizing any substrate and applied towards the production of any product.

## Background

Microorganisms are increasingly utilized to synthesize a variety of products [1-3], including fuels (bio-alcohols [4-13] and biodiesels [14,15]), specialty chemicals (amino acids [16-20]), therapeutic small-molecules [21-25] (antibacterials, anti-cancer agents, and cholesterol-lowering agents), and biopharmaceuticals [26] (proteins, vaccines, and virus particles). A common challenge in developing high-yield cellular production systems is that organisms have evolved to optimize growth rather than the formation of a particular end-product. In principle, this challenge could be met by reprogramming the cellular objective using genetic modifications (such gene insertions, over-expressions, or deletions). In practice, the selection of appropriate gene modification targets can be a daunting task. Biomass formation as well as product synthesis requires building block precursors and cofactors provided through the concerted actions of a large number of interconnected metabolic pathways encoded by hundreds to thousands of genes. While purely empirical attempts at genetic modifications have in some cases led to impressive success [27], these cases have provided the exceptions rather than the rule. There is now considerable evidence that substantial improvements in productivity require manipulating the activities of multiple enzymes in different parts of cellular metabolism [28]. In this respect, optimizing biosynthetic productivity will almost certainly benefit from computational modeling tools that systematically and efficiently explore the consequences of gene- or enzyme-level modifications across the breadth of cellular metabolism.

Currently, there exists a variety of methods for studying metabolic networks in both quantitative and qualitative manners: flux balance analysis (FBA) [29-31], ^13^C-labeling based metabolic flux analysis (^13^C-MFA) [32], metabolic control analysis [33], elementary mode analysis (EMA) [34], extreme pathway analysis [35], cybernetic modeling [36,37], and biochemical systems theory [38-40]. Many of these methods do not necessarily identify experimentally tractable metabolic engineering targets such as gene deletions. Whereas, some algorithms based on the aforementioned methods can be used to identify such targets including minimization of metabolic adjustment (MoMA) [41], regulatory on/off minimization (ROOM) [42], OptKnock [43], OptStrain [44], OptReg [45], and OptGene [46]. All six of these methods require solving an optimization problem to determine flux distributions as a means of evaluating the strain's (or mutant strain's) metabolic capabilities. Although these optimization approaches can accurately predict optimal growth and production fluxes in some cases [47], other experimental settings produce inaccurate predictions [48]. In addition, situations that require the removal of numerous genes to achieve high productivity will lead to mutant strains significantly different from wild-type systems, further weakening the assumptions behind FBA. OptKnock and OptStrain utilize a bi-level optimization for determining superior mutant strains. The mixed integer linear programming (MILP) framework used in these two algorithms optimizes for one objective within another competing one (a cellular objective (biomass production) within an engineering objective (chemical production)). However, the user must provide the number of knockouts that OptKnock and OptStrain can allow. In general, exhaustively searching genomic space for knockout candidates is computationally intractable even on small-scale metabolic models (less than 100 reactions), much less on current genome-scale metabolic models (greater than 1000 reactions) due to prohibitive computation time. This situation coupled to the fact that two or three knockouts are likely not sufficient for generating a mutant capable of maximal productivity motivated the use of a genetic algorithm as demonstrated in OptGene.

Genetic algorithms (GAs) have been classically utilized as a search method for optimization of objective functions that are discontinuous, non-differentiable, stochastic, or highly non-linear. As the name implies, the underlying theory behind GAs is based on Darwinian evolution. GAs seek to evolve a population of potential solutions by crossover and mutation, and through multiple generations, the entire population will eventually "evolve" towards a global optimum (a "fitness score"). They have been used to some extent in modeling biological systems [46] and are used as a search technique in this study. Though OptGene utilizes a GA to efficiently explore genotypic space, the framework still requires the use of a metabolic assumption required to determine metabolic fluxes (such as FBA, MoMA, or ROOM). The goal of this study was to leverage the power of a GA without the need for a metabolic assumption. As such, a primary objective of this work was to identify a genotype with a high productivity phenotype *strictly *from the wild-type organism's metabolic network topology, utilizing thermodynamics.

An elementary mode (EM) is a non-decomposable set of reactions (encoded by a set of genes) that leads to a functional metabolic pathway. EMA is a method of enumeration of all of the EMs of a metabolic network. As such, an EMA presents a convex analysis problem from computational geometry, in which the extreme rays of the polyhedral cone (as defined by stoichiometry and reversibility) are the EMs of the metabolic network. As a result, an EM represents a single functional pathway within overall cellular metabolism, a linear combination of a cell's EMs can be used to describe *any *metabolic state achievable by the cell's stoichiometry. The algorithmic complexity of this problem has not been studied in detail and has therefore been classified as at least an NP-hard (non-deterministic polynomial-time hard) problem [35]. Empirical observations have shown that the computation time of EMA algorithms grows approximately quadratically with respect to the number of EMs, and unfortunately, the number of EMs grows exponentially with respect to network size. As a result, the computation time increases greatly with respect to network size, limiting analysis to non genome-scale metabolic networks. Nonetheless, EMA has been utilized to design strains of *E. coli *that are efficient at producing biomass from glucose [49] and ethanol from five- and six-carbon sugars [50]. In two cutting-edge applications, EMA was combined with linear programming to determine flux distributions from external measurements in lysine-producing *Corynebacterium glutamicum *[51], and to determine the metabolic fluxes of *Lactobacillus rhamnosus *growing on medium containing mixed substrates [52]. EMA has also been utilized to determine flux distributions in polyhydroxybutyrate-producing *E. coli*, mediated by a thermodynamic analysis of the EMs [53].

In this study, an algorithm based on EMA, pathway thermodynamics, and a GA has been constructed with the goal of redesigning a metabolic network towards maximally producing compounds of interest while simultaneously sustaining high biomass formation. This algorithm was applied to producing ethanol and heterologous lycopene through *E. coli*. We addressed issues of computation speed by coupling the EMA model with a GA to efficiently explore genotypic space. This algorithm presents a combination of a variety of traits that have been explored previously by themselves: 1) it is based solely on reaction stoichiometry and network topology, independent of any experimental flux measurements (although, if flux measurements are available, they can be used to constrain the problem); 2) by the utilization of a GA, it contains an efficient search method arriving at a solution within minutes on a single-processor notebook system; 3) by the utilization of a GA, it arrives at an optimal solution in a computationally tractable amount of time; and 4) it contains the option to use a multi-objective genetic algorithm (MOGA), only utilized very recently in analysis of metabolic networks, but still within the context of FBA [54].

## Results & Discussion

### Algorithm

**Figure 1:**
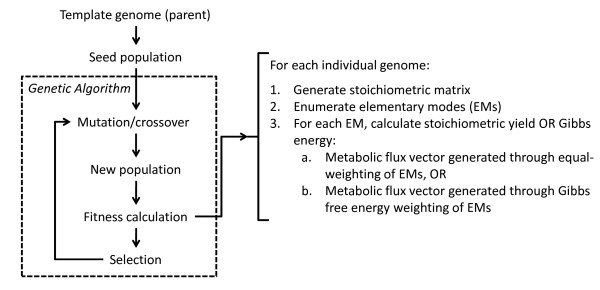
**Schematic overview of the framework**. The following optimization objectives (fitness functions) were considered: (*a*) calculating metabolic fluxes through equal-weighting of the EMs, (*b*) calculating metabolic fluxes through weighting the EMs based on their corresponding Gibbs free energy cost, and (*c*) minimizing the average Gibbs free energy cost of EMs forming both product and biomass. The free energy cost of a reaction route was calculated as the stoichiometric sum of its reaction Gibbs energies. Reaction routes with net negative energy costs are considered favorable.

The framework is schematically shown in Figure 1. A GA is used for the optimization, by evolving a population of potential solutions (strains) towards the global optimum solution (the theoretical yield of either product or biomass on substrate). A strain is represented by a binary vector, or a chromosome, where an entry of "1" indicates a particular reaction corresponding to the position of the entry included in the strain and a "0" indicates that the reaction is not present. Each strain is then evaluated based on a fitness criterion. The strain's EMs are then enumerated and its corresponding metabolic flux vector is generated by taking a weighted linear combination of the EMs in two ways: 1) equal weighting of the EMs, or 2) weighting the EMs based on their corresponding Gibbs free energy associated with the pathway (Δ*G*_*p*_°). Next, the yields of biomass and product on substrate are calculated and the strain's corresponding fitness is evaluated. This fitness, a reflection of the cellular phenotype, is then used by the GA to enrich the population of potential solutions to those that have a higher fitness value. This process is repeated until one of the GA's stopping criteria is met.

### Elementary Mode Analysis

**Figure 2:**
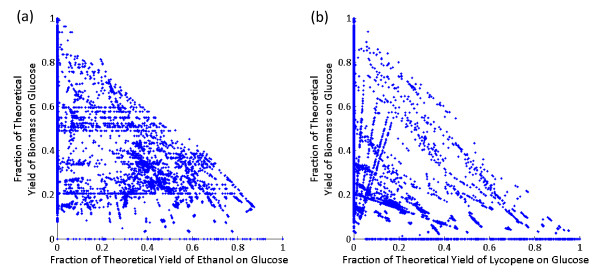
**Product vs. biomass yield for every EM in the (*a*) ethanol and (*b*) lycopene model**. The number of EMs was 33,220 and 42,659 for the ethanol and lycopene models, respectively (more information on these models can be found in Table 1). Yields are expressed as fractions of the theoretical yield of product or biomass. The theoretical yields of product or biomass on substrate were determined by searching the EMs of the template-genome (the parent strain) and finding the maximal yields for product or biomass on substrate, respectively. Each point in the scatter plot corresponds to an EM (or number of EMs) with a pair of product and biomass yield values. Several EMs were associated with the same pair of product and biomass yield values, which are represented by a single point.

As test cases, two small-scale stoichiometric models were constructed of ethanol- or lycopene-producing *Escherichia coli *using a previously published model as a template [50], the details of which can be seen in Table 1. The dimensions of the ethanol and lycopene models were 47 × 60 (47 metabolites and 60 reactions) and 50 × 64 (50 metabolites and 64 reactions), respectively. The ethanol model supported 33,220 EMs: 8,389 (25.3%) of which produce ethanol, 28,336 (85.3%) of which produce biomass, and 7,156 (21.5%) of which produce both ethanol and biomass. The lycopene model supported 42,659 EMs: 9,439 (22.1%) of which produce lycopene, 32,763 (76.8%) of which produce biomass, and 4,427 (10.4%) of which produce both lycopene and biomass. A scatter plot (Figure 2) of each EM's product vs. biomass yield showed a negative correlation for both models, suggesting a pattern of trade-off between cell growth and product formation. This trade-off was most evident for EMs with either very high biomass or product yields. The theoretical yield of ethanol on glucose is 0.511 g g^1 ^and the theoretical yield of lycopene on glucose is 0.316 g g^1^. There are 14 (0.04%) EMs that produce the theoretical yield of ethanol on glucose (requiring between 39 and 44 reaction removals), while there is only one EM (0.002%) that produces the theoretical yield of lycopene on glucose (requiring 36 reaction removals). EMA was conducted on a notebook equipped with an Intel^® ^Core™ 2 Duo T9300 CPU running at 2.50 GHz, 4.0 GB memory, and a 32-bit version of Microsoft Windows Vista™ Ultimate. The computation time required to enumerate the entire set of EMs was 8.04 s ± 0.27 s (*n *= 10) and 12.30 s ± 0.24 s (*n *= 10), for the ethanol and lycopene models, respectively. More detailed information of both models can be found in *Additional File *1 and *Additional File *2.

**Table 1:** Information on the ethanol and lycopene models, their corresponding EMs, and their reaction and pathway change in Gibbs free energy.

	Ethanol Model	Lycopene Model
**Number of reactions**	60	64
**Number of metabolites**	47	50
**Mean EMA Computation Time (s) (± SD)**	8.04 s ± 0.27 s	12.30 s ± 0.24 s
**Number of EMs**	33,220	42,659
**Number of product-producing EMs (%)**	8,389 (25.3%)	9,439 (22.1%)
**Number of biomass-producing EMs (%)**	28,336 (85.3%)	32,763 (76.8%)
**Number of product- and biomass-producing EMs (%)**	7,156 (21.5%)	4,427 (10.4%)
**Δ*G*_*r*_° Mean (kcal mol^1^)**	1.95	1.77
**Δ*G*_*r*_° Skewness (kcal mol^1^)**	4.54	0.42
**Δ*G*_*p*_° Mean (kcal mol^1^)**	111.87	88.72
**Δ*G*_*p*_° Median (kcal mol^1^)**	71.71	50.40
**Number of thermodynamically-infeasible EMs (%)**	242 (0.73%)	1487 (3.49%)

### Pathway Gibbs Free Energies

**Figure 3:**
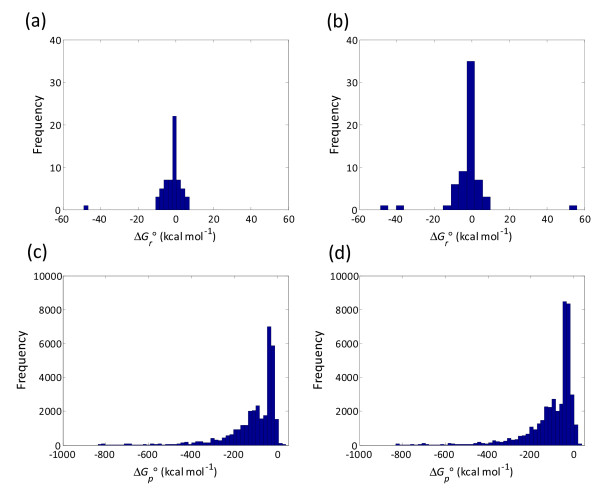
**Histogram of reaction Gibbs free energies for the (*a*) ethanol and (*b*) lycopene models**. Also plotted is a histogram of the EM Gibbs free energies for the (*c*) ethanol and (*d*) lycopene models. Gibbs free energies for the EMs were computed as described in the text.

Standard Gibbs energies of formation (Δ*G*_*f*_°) of metabolites included in the two models were calculated using group contribution theory [55] from previously reported data [56]. These values were used to estimate the Gibbs energy changes of reactions in the model. Technically, these estimates correspond to Gibbs energy changes defined for standard conditions (Δ*G*_*r*_°) (298.15 K, 1 atm, pH 7.0, all compounds at 1 M), rather than physiological conditions. Consequently, it is quite likely these estimates deviate slightly from experimentally determined values. In this study, we used these estimates as first-order approximations to derive the Gibbs energy changes across metabolic pathways (Δ*G*_*p*_°) as defined by the EMs. Histograms of Δ*G*_*r*_° and Δ*G*_*p*_° values (Figure 3) show qualitatively different distributions. The Δ*G*_*r*_° histogram approximates a normal distribution about zero; whereas, the Δ*G*_*p*_° histogram clearly skews in the negative direction. The mean Δ*G*_*r*_° values for both models are slightly negative (Δ1.95 kcal mol^1 ^and 1.77 kcal mol^1 ^for the ethanol and lycopene models, respectively). Interestingly, the skewness of both distributions is different: the ethanol model has a negative skewness of 4.54 kcal mol^1 ^while the lycopene model has a slight positive skewness of 0.42 kcal mol^1^. This indicates a change in distribution to slightly more energetically favorable reactions with respect to the ethanol model. The mean values for Δ*G*_*p*_° are much more negative at 111.87 kcal mol^1 ^and 88.72 kcal mol^1 ^for the ethanol and lycopene models, respectively. Interestingly, the ethanol model contains relatively few (242, only 0.73%) thermodynamically infeasible EMs (having Δ*G*_*p*_° > 0), while the lycopene model contains both a higher number of thermodynamically infeasible EMs in both number (1487) and percentage (3.49%). This exemplifies the nature of a host engineered to produce a compound it does not normally produce, showing that a variety of the pathways are not evolved.

Previous analyses have shown that most metabolic reactions are near equilibrium (|Δ*G*_*r*_|≈ 0) [57]; whereas, metabolic pathways are energetically favorable (Δ*G*_*p *_< 0) [56], consistent with the trends shown in the panels of Figure 3. In this regard, the calculated Δ*G*_*p*_° values should offer qualitatively correct and quantitatively reasonable estimates of the thermodynamic favorability of metabolic pathways. The change in Gibbs free energy across an entire pathway (an EM) is much more likely to be negative than the change in Gibbs free energy across individual reactions within the pathway as shown in Figure 3c and Figure 3d. Therefore, there is a strong correlation between stoichiometric feasibility and energetic favorability at the level of the pathway. Moreover, thermodynamic favorability has already been used in the past to narrow the solution space or eliminate infeasible solutions when estimating or optimizing metabolic flux distributions [57,58]. In the algorithm, we used the Δ*G*_*p*_° estimates to identify thermodynamically favorable reaction routes and enrich the mutant organism with these favored routes towards product and biomass production.

As stated previously, cellular metabolism (a flux vector) can be represented as a linear combination of the cell's EMs. It has been demonstrated previously that there exists a strong correlation between the standard change in entropy across an EM (Δ*S*_*p*_°) and the weighting factor of its contribution to the overall flux state in both a wild-type *E. coli *strain and a strain engineered for polyhydroxybutyrate production [53]. The resulting method of determining fluxes based on weighting by Δ*S*_*p*_° values was then compared to flux values reported in literature and showed a strong correlation (*R*^2 ^= 0.85). In an analogous manner, we utilized Gibbs free energies for flux determination similar to previous efforts [57].

### Comparison of Calculated Fluxes with Alternative Methods

**Figure 4:**
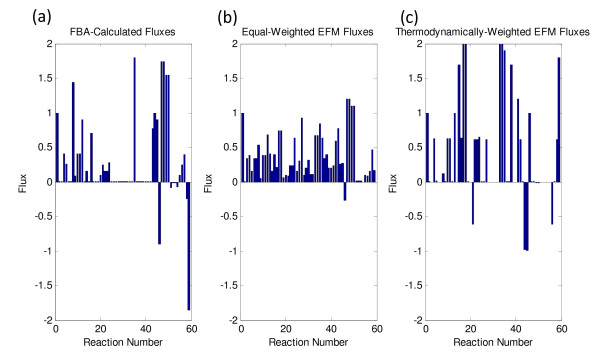
**A bar plot of fluxes for the ethanol model calculated using (*a*) FBA linear optimization, (*b*) equally-weighted EMs, and (*c*) thermodynamically-weighted EMs**.

The flux profiles of the *E. coli *model (without the additional lycopene biosynthetic reactions) using both equal-weighting of the EMs and thermodynamic weighting of the EMs were compared to fluxes calculated by FBA (Figure 4). In the linear programming approach of FBA, fluxes are determined through the utilization of an objective function which maximizes the biomass equation ("growth-rate"). This is an inherently different approach in which optimization is utilized to determine the flux distribution; whereas, the approach in this algorithm only uses optimization for redesigning the cellular genotype. As such, the algorithm's flux-determination method makes no metabolic assumptions; instead, it assumes that the flux distributions are determined by well-grounded thermodynamic principles, which have been shown to be accurate in previous studies [53].

### Ethanol Case Study

Two case studies were performed to evaluate our algorithmic framework. In these case studies, the algorithm was tasked to identify gene knockouts that would result in the optimal product yield, biomass yield, or overall productivity (biomass yield × product yield). The two cases were: aerobic production of ethanol (a native compound) and aerobic production of lycopene (a heterologous compound), both in *E. coli*. The search space for the algorithm excluded reactions that computationally led to either no biomass formation or no product formation. For the ethanol case study, these reactions were BIO, FEM5, FEM6, GG1, PPP5r, TCA1, TCA2r, TCA3r, TCA4, TRA1, and TRA3 (see *Additional File *1 for more information). These reactions were identified by conducting a single reaction removal analysis on the wild-type model. The reactions were removed individually, the EMs were enumerated, and the EMs for each mutant were rank ordered based on their stoichiometric ethanol yield or biomass yield. If the maximal yield for either ethanol or biomass was zero, then these reactions were considered to be necessary. As a result, any strain that contained any one of these knockouts would produce either no ethanol or no biomass.

**Figure 5:**
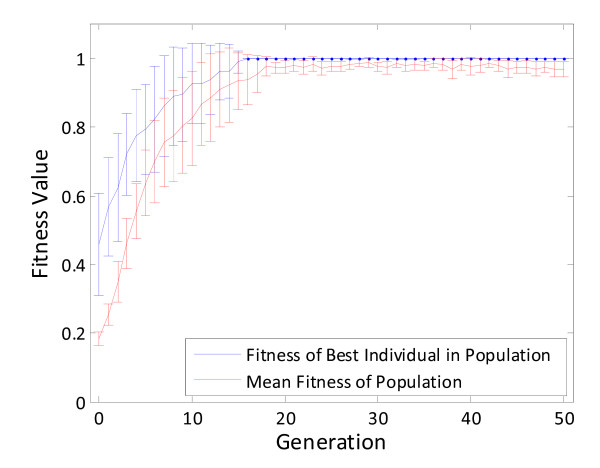
**A plot of average and mean fitness scores (± one standard deviation, *n *= 10) as a function of generation number for optimizing ethanol production using equally-weighted EMs**.

In this case study, equal weighting of the EMs and weighting the EMs based on their corresponding Gibbs free energies led to fitness values of 1.000 for ethanol production (the fitness function described in Eq. 5) requiring 13 and 11 reaction removals, respectively. This indicates that ethanol production at the theoretical yield is thermodynamically feasible. The fitness values for the coupled ethanol and biomass production fitness function were similar, though not identical: 0.266 (equal weighting) and 0.241 (thermodynamic weighting). The individual yields of ethanol and biomass were slightly different, with the ethanol fractional yield being higher in both cases. This highlights a potential advantage of utilizing a MOGA: the array of solutions will display a range of organism phenotypes that include: 1) high-producing, slow-growing, 2) low-producing, fast-growing, and 3) moderate-producing, moderate-growing. In most of the cases investigated here, a coupled fitness function (Eq. 7) leads to organisms generated by the third case. However, the potential in using a MOGA is the ability to choose what type of organism the researcher would like to construct based on ease of construction or process economics. As can be seen in Figure 5, the algorithm as formulated here is quite robust at finding the global optimum quickly, even given varying initial conditions.

Whenever ethanol was being optimized (either by itself or with biomass), reactions for either NADH dehydrogenase I/ATP synthase (OPM1) or NADH dehydrogenase II (OPM4r; also known as NADH:ubiquinone oxidoreductase II) were removed. Both NADH dehydrogenase I and II are involved in driving electron flow, while NADH dehydrogenase I is driven by oxygen. NADH dehydrogenase II uses NADH exclusively and is repressed when *E. coli *is grown anaerobically [59]. The results predicted using either EM-weighting method are consistent with what is known about ethanol production through *E. coli*, namely, that it is mainly produced anaerobically. Also for the majority of the cases, reactions for the pyruvate oxidase (coded by *poxB*) and phosphate acetyltransferase (coded by *pta*) were identified for removal, consistent with what has been previously reported for improving ethanol production from glucose through *E. coli *[49]. Depending on the weighting scheme, reactions identified for removal were fumarate reductase (coded by *frdABCD*), malate dehydrogenase (coded by *sfcA *and *maeB*), or lactate dehydrogenase (*ldhA*), all also identified by the pervious study as a near optimal producing genotype.

All of the solutions generated strains with between 16 and 24 EMs (over a three order of magnitude reduction in EMs); the different fitness functions produced mutant genomes that shared a number of similarities. Across many of the solutions, reactions in oxidative phosphorylation (as previously described) were removed. Many of the fermentative acid pathways were also removed (most notably for acetate and lactate production), which would limit the formation of undesired byproducts. Relative few reactions in glycolysis, the TCA cycle, and the PPP were removed in the different weighting schemes. It is also interesting to note that the main fermentative pathway preferred by *E. coli *(the acetic acid pathway) and the lactic acid pathway are chosen for removal over other secreted weak acid metabolites (formate, succinate, and pyruvate). It is therefore likely that the removal of other fermentative pathways would improve flux to the ethanologenic pathway. Taken together, the general results across many of the fitness functions suggest removal of certain fermentative and anapleurotic pathways improves cellular phenotype.

As stated, many of the knockouts identified by the algorithm presented here have been reported in *E. coli*. The *frdA *and *ndh *knockouts were also identified by an EMA-based algorithm and implemented in the laboratory to improve ethanol production [50]. The *nuo *and *atp *operons were neglected in the previous model because the model was restricted to anaerobic action, therefore as stated previously, the algorithm here correctly identifies these as knockout targets (akin to operating anaerobically). However, the *nuo *knockout increases glucose uptake and ethanol production, while decreasing acetate, succinate, lactate, and formate formation in an anaerobic chemostat with complex medium supplemented with glucose [60]. While the previous algorithm identified a variety of other knockouts predicted to improve production, the algorithm design was slightly different and exemplifies one of the challenges in both engineering and modeling biological systems. As has been shown here and in other places [61,62], multiple genotypic states can lead to the same phenotypic state.

### Lycopene Case Study

Next, the *E. coli *metabolic network was optimized for lycopene production. Lycopene is a C_40 _carotenoid natural product with antioxidant properties. Much work has been devoted to engineering lycopene biosynthesis in *E. coli *[63-72], due to the fact that it shares metabolic precursors (DMAPP and IPP) to other isoprenoid natural products with immense therapeutic value, such as the antimalarial sesquiterpene artemisinin and the anticancer diterpenoid paclitaxel [70].

Using the same approach as in the ethanol case study, the results were slightly different here. In general, the solutions generated strains with more EMs (between 18 and 174), perhaps due to the fact that the number of EMs in the parent network was nearly 30% larger for this model. First, the thermodynamically-weighted cases often converged to lower values than the equal-weighted cases when lycopene yield was included in the fitness function. Second, the fitness value for both the product yield and biomass yield optimization cases were suboptimal (less than one). While this was the case for biomass production in the ethanol model, it was not the case for product formation. This is likely due to the fact that the ethanol model had 14 EMs capable of producing the theoretical yield of product on glucose, while the lycopene model only had 1 EM that produced the theoretical yield. This genotype resulted in 36 reaction removals, which is well above the number of reaction removals found in the optimal solutions presented in Table 2 and Table 3. In both cases, the algorithms were run multiple times to confirm the result, and the same fitness values were achieved. This result may be due to the fact that the original problem was seeded with an initial population of individuals containing between two and six removals, which may bias solutions with fewer knockouts (which is certainly experimentally amenable). This could also be due to the severe drain of the cellular NADPH pool required for lycopene biosynthesis (the pathway for lycopene biosynthesis requires 16 molecules of NADPH consumed for 1 molecule of lycopene produced) [64].

**Table 2:** Information on resulting strains after running the algorithm for the ethanol model.

	Equal-Weighted			Thermodynamically-Weighted		
Fitness function^a^	Ethanol	Biomass	Ethanol × Biomass	Ethanol	Biomass	Ethanol × Biomass
**Number of reactions removed**	13	10	18	11	10	11
**Number of associated genes (or gene clusters)^b^**	16	8	18	12	10	9
**Gene list**	*aceA* *ackAB* *atpABCDEFGHI* *cyoABCD* *fbp* *frdABCD* *glpX* *ldhA* *mdh* *nuoAHJKLMNEFGBC* *pflB* *poxB* *pta* *talAB* *tdcE* *tktAB*	*aceA* *aceEF* *ldhA* *lpdA* *mdh* *pgi* *pntAB* *tktAB*	*adk* *atpABCDEFGHI* *cyoABCD* *eda* *fbp* *frdABCD* *glpX* *ldhA* *maeB* *mdh* *ndh* *pckA* *pflB* *poxB* *pps* *pta* *sfcA* *tdcE*	*atpABCDEFGHI* *cyoABCD* *fbaAB* *gnd* *maeB* *mdh* *ndh* *poxB* *pta* *rpe* *sfcA* *sucCD*	*atpABCDEFGHI* *cyoABCD* *frdABCD* *maeB* *pgmA* *pgml* *pntAB* *sfcA* *tktAB* *ytjC*	*aceA* *adk* *atpABCDEFGHI* *cyoABCD* *eda* *ndh* *pntAB* *pta* *sucCD*
**Total number of EMs**	23	23	23	16	17	24
**Biomass yield (fraction of theoretical)**	0.000	0.839	0.456	0.000	0.861	0.483
**Ethanol yield (fraction of theoretical)**	1.000	0.007	0.584	1.000	0.003	0.499

^a ^Fitness functions are defined in the Methods section. ^b ^Enzymes with multiple subunits encoded by different genes in the same operon are only counted once.)

**Table 3:** (Information on resulting strains after running the algorithm for the lycopene model.

	Equal-Weighted			Thermodynamically-Weighted		
Fitness function^a^	Lycopene	Biomass	Lycopene × Biomass	Lycopene	Biomass	Lycopene × Biomass
**Number of reactions removed**	17	14	13	10	12	10
**Number of associated genes (or gene clusters)^b^**	21	15	12	9	9	10
**Gene list**	*aceA* *adhE* *atpABCDEFGHI* *cyoABCD* *eda* *fbp* *fdhF* *frdABCD* *fumABC* *glpX* *hycBCDEFG* *ldhA* *maeB* *ndh* *aceEF* *pflB* *ppc* *pps* *pykAF* *sfcA* *tdcE*	*ackAB* *adhE* *adhP* *frdABCD* *lpdA* *maeB* *ndh* *aceAB* *pckA* *pgi* *poxB* *sfcA* *sucAB* *sucCD* *talAB*	*ackAB* *adhE* *adhP* *adk* *fbp* *fdhF* *glpX* *hycBCDEFG* *ldhA* *pckA* *ppc* *pta*	*adk* *eda* *frdABCD* *gnd* *ndh* *aceB* *rpe* *sucCD* *tktAB*	*aceA* *adhE* *adhP* *fbaAB* *ldhA* *maeB* *ndh* *rpe* *sfcA*	*aceAB* *adhE* *atpABCDEFGHI* *cyoABCD* *ldhA* *pgmA* *pgml* *poxB* *pta* *ytjC*
**Total number of EMs**	18	21	80	174	35	86
**Biomass yield (fraction of theoretical)**	0.000	0.900	0.272	0.000	0.842	0.410
**Lycopene yield (fraction of theoretical)**	0.968	0.015	0.698	0.646	0.001	0.418

^a ^Fitness functions are defined in the Methods section. ^b ^Enzymes with multiple subunits encoded by different genes in the same operon are only counted once.)

A pioneering study on applying computational methods (specifically, MoMA) for driving metabolic engineering studies focused on lycopene production from glucose minimal medium through *E. coli *[64]. The single knockout target search identified seven knockout targets (*gdhA*, *cyoA*, *gpmAB*/*yjtC*, *ppc*, *glyA*, *eno*, and *aceE*), two of which are not included in the model used here as they pertain to amino acid metabolic pathways (*gdhA *and *glyA*). Of the other five, all were identified in some capacity by our model when optimizing for lycopene or lycopene and biomass, some of them in multiple cases. Of these computationally identified knockouts, numerous single-, double-, and triple-knockout strains were constructed in the laboratory and showed improved lycopene-producing phenotypes.

The *fdhF *knockout (identified here as a knockout candidate in the equal weighting cases, both with and without biomass production) improved specific lycopene production by 4%. Combining these two knockouts with a *gdhA *knockout (as identified by genome-scale MoMA simulations, but not considered in the model presented here) resulted in the best triple knockout strain, improving specific lycopene production by 37% [64]. The *nuo *knockout improved specific lycopene production by 45% (from 1,100 ppm to 2,040 ppm) in complex medium supplemented with glucose. The other knockouts identified in this case have either not been reported with respect to improving lycopene production, or are lethal to the cell (as is the case with *pgk*). The *aceE *knockout was identified by MoMA simulations and implemented in the laboratory, improving specific lycopene production by 9% in minimal medium supplemented with glucose [64]. The *pykAF *double-knockout improved specific lycopene production three-fold (from approximately 5 to 15 mg gDCW^-1^) in complex medium [69]. While many of these knockouts have not been conducted in the same strain, there remain many opportunities to improve lycopene titers. Currently, lycopene production yields reported are well below the theoretical yield on glucose (316 mg/g glucose). For example, bioreactor cultivation of the over-producing *ΔgdhA ΔaceE ΔfdhF *triple knockout strain resulted in a lycopene yield of 2.15 mg g glucose^-1 ^[66], less than 1% of the theoretical yield. It is reasonable to assume that numerous additional knockouts would further aid efforts to reach this theoretical yield. Overall, the reported literature on metabolic engineering effort to improve lycopene production in *E. coli *strongly supports the validity of the algorithm developed here.

### Multiobjective Genetic Algorithm

Given the dual objective nature of the system in question (product yield and biomass yield), it would be logical to also assess the performance of a multi-objective genetic algorithm (MOGA). MOGA maximizes/minimizes a vector of objective functions (in this case, a vector of length two) rather than a scalar objective, as was the case for the GA. As a result, there is no single, unique solution to this problem. Instead of identifying a single solution, a MOGA aims to identify a set of solutions in which an improvement in one objective requires a decrease in the other. Each solution is considered to be a non-inferior solution and the entire set of non-inferior solutions is referred to as the Pareto optima. The MOGA invoked here uses a controlled elitist genetic algorithm, a variant of the Non-dominated Sorting Genetic Algorithm-II (NSGA-II).

**Figure 6:**
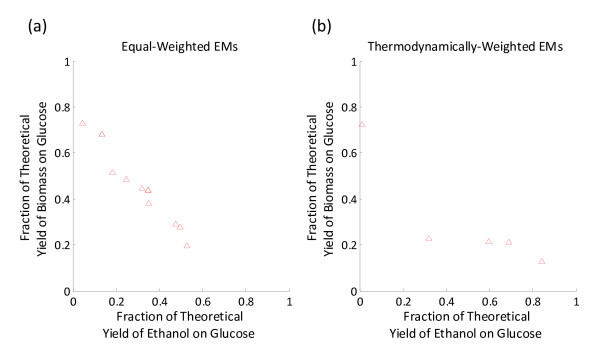
**A plot of the Pareto optima and the fitness values for the corresponding non-inferior solutions for the ethanol model using (*a*) equally-weighted EMs and (*b*) thermodynamically-weighted EMs**.

The multi-objective version of the algorithm was tested on the ethanol model, with both equal- and thermodynamic-weighting of the EMs. In each case, of the entire final population, only a small fraction of the individuals (<10%) had non-inferior solutions. In the case for equal-weighting of the EMs, the best individuals had fitness values of 0.3475 and 0.4375 (for Eq. 10 and Eq. 11, respectively), which has a product of 0.1520 and is much lower than the value of approximately 0.25 found in the solution of the single-objective GA with the coupled fitness function (Eq. 7). Similarly, while thermodynamically-weighting the EMs, the best individual in the Pareto optima had fitness values of 0.6885 and 0.2112 and a product of 0.1454, also much lower than the value of approximately 0.26 found in the solution of the single-objective GA with the coupled fitness function. As stated previously, an advantage of the MOGA is that it allows the user to "choose" whether to pursue constructing a strain predicted to have slightly lower growth rate but higher product yield versus a strain predicted to have a slightly higher growth rate but lower product yield (as can be seen in the scatter plots in Figure 6). However, in this study, the suboptimal values of the fitness functions and increased computational times place the MOGA approach at a disadvantage when compared to the single-objective GA.

Though MOGAs have not been used for optimizing the structure of metabolic networks, there has been a recently reported example of using one for optimizing an industrial bioprocess (penicillin V production from *Penicillium chrysogenum*) [73]. In particular, a MOGA was used for 1) maximizing penicillin titer and maximizing penicillin yield from substrate, 2) maximizing penicillin titer and minimizing fermentation time, among other decision variables. While all of these were optimizing for two objectives, the authors invoked a tri-objective GA yield for simultaneously optimizing penicillin titer, penicillin yield, and profit.

## Conclusions

This article presents the development and application of a computationally tractable framework which combines elementary mode analysis, pathway thermodynamics, and a genetic algorithm. The framework was then used to efficiently redesign the *E. coli *metabolic network for maximal production of two industrially-relevant products, ethanol and lycopene. Our results show that *E. coli *metabolism can be re-tailored quite efficiently for optimal or near-optimal production of a product of interest (ethanol or lycopene were examples here), biomass, or coupled product and biomass. As discussed, many of the gene knockouts identified by the algorithm to improve production formation have been tested experimentally (however, most often individually and not in combination) and have been shown to improve product formation rates.

It has been shown that the contribution of an individual EM to overall cellular metabolism can be estimated from its pathway thermodynamics [53]. It has been proposed that this is a result of billions of years of evolution underlying the metabolic regulation and expression patterns of the genes within these pathways. As a result of this proposal, it can be assumed that a cell will attempt to reduce its overall free energy by favoring pathways (EMs) that have a more negative Gibbs free energy. Equivalently, pathways with a positive free energy are thermodynamically infeasible and are not assigned a weight in the analysis presented here (for the case of thermodynamic weighting). This allows flux determination based solely on reaction stoichiometry and thermodynamics from the EMs generated by EMA, rather than applying a metabolic assumption (maximizing growth rate) in optimization based studies (such as FBA). It is important to note that these weighting factors are not strictly predetermined but are determined *within *the context of the overall cellular network.

Generally speaking, equal-weighting of the EMs was shown as a proof-of-principle demonstration of the algorithm. At the same time, this was used as a reference to determine whether certain gene knockouts were predicted under both weighting schemes. Ideally, a significant fraction of the gene knockouts identified would be consistent between equal and thermodynamic weighting of the modes. As a result, the incorporation of thermodynamic calculations was an integral part of this algorithm providing for more accurate flux distributions (as compared to FBA-calculated fluxes).

The utilization of a GA to search the solution space enables the identification of an optimal genotype in a computationally tractable amount of time. The number of reaction removals required to meet these predicted optimal values are well above what is computationally feasible through exhaustive searching. For example, even ten reaction removals (the smallest number for the ethanol case study) would require evaluating 2.74 × 10^17 ^*in silico *organisms. With the genetic algorithm, the simulations here converged when evaluating only 2,500 *in silico *organisms (50 generations of 50 individuals).

Metabolic and genetic networks are highly connected with significant regulation across scales, even for microbial systems. A clear disadvantage of the model and algorithm presented, as well as most of stoichiometric modeling, is the lack of integrated regulatory information. Because these models are used to study steady-state behavior, the dynamic regulation of these systems is neglected. There have been efforts to reconstruct genome-scale transcriptional and translational (TR-TR) networks and transcriptional regulatory networks (TRNs) [74,75]; however, the integration of these models with metabolic models has been somewhat limited [76-79]. Utilizing EMA for identifying knockout targets for improving ethanol production in *E. coli *allowed for simultaneous utilization of pentoses and hexoses in batch culture [50]. This shows that a strictly stoichiometric analysis using EMA can synthetically de-regulate catabolite repression (perhaps the most well-studied means of metabolic regulation).

A potential limitation of this method is the utilization of EMA, which is computationally intensive and currently cannot be applied to genome-scale metabolic networks. As cited previously, the computation time of EMA algorithms grows approximately quadratically with respect to the number of EMs and the number of EMs grows exponentially with respect to network size. For example, an *E. coli *model of 110 reactions (28 of which were reversible) using any combination of glucose, succinate, glycerol, and acetate contained 507,632 EMs [80]. However, when making many small-molecule products through *E. coli*, minimal medium with a single carbon-source is often used such that many of the reactions in *E. coli *metabolism would not acquire flux. Therefore, the engineering of high-flux pathways (glycolysis, the TCA cycle, etc.), as represented in this small-scale model, would have more impact on product formation. Very recently, the concept of elementary flux patterns was introduced, where an elementary flux pattern is defined as a set of reactions within a subsystem of a larger network that represents the basic routes of each steady-state flux of the larger network through the sub-network [81]. They are computed using MILP and as a result, this technique can be applied to genome-scale networks, a quality mediated by the fact that computation time climbs only polynomially with respect to network size. Also very recently, an algorithm was developed to identify the *K*-shortest EMs within a genome-scale metabolic network utilizing integer linear programming [82]. The algorithm here could be similarly applied to these two recently developed algorithms.

## Methods

### Model Construction

The two small-scale *E. coli *stoichiometric models utilized in this study were based on one previously developed [50]. Briefly, because the previous model was developed for the utilization of multiple five- and six-carbon sugars, all of the carbon-source utilization reactions besides the glucose utilization reaction were removed; glucose was assumed to be actively uptaken by the phosphoenolpyruvate sugar transferase system. In the original model, an additional reaction was included due to a heterologous pyruvate decarboxylase from *Zymomonas mobilis*; this reaction is not native to *E. coli *and was therefore also removed.

For the lycopene case study, the ethanol model previously described served as a basis with four additional reactions added. Lycopene biosynthesis was introduced into the model and coupled to the non-mevalonate pathway (native to *E. coli*) previously used to support heterologous carotenoid production [83,84]. Whenever possible, linear pathways were combined into a single reaction to reduce the size of the model. The first reaction (encoded by *dxs *and *ispCDEFGH*) held the stoichiometry: glyceraldehyde-3-phosphate + pyruvate + 2 NADPH + ATP → dimethylallyl diphosphate (DMAPP) + CO2 + 2 NADP^+ ^+ ADP. The second reaction was for the reversible isomerization of DMAPP and isopentyl diphosphate (IPP), encoded by *idi*. The third reaction held the stoichiometry 4 IPP → geranylgeranyl diphosphate (GGPP) and is encoded by *ispA *and *crtE*. The last reaction was for lycopene biosynthesis and held the stoichiometry 2 GGPP + 8 NADPH → lycopene + 8 NADP^+^, and is encoded by *crtBI*. To avoid the inclusion of a specific transport reaction, lycopene was not balanced in this reaction; however, it was taken into account for thermodynamic calculations. The final model included three more metabolites and four more reactions than the ethanol model.

### Elementary Mode Enumeration

Elementary mode analysis (EMA) was undertaken utilizing the bit pattern tree method [85]. Developed recently, this algorithm is capable of enumerating 2,450,787 EMs over ten-times faster (on a four-thread system) than the latest release of METATOOL [86], and is therefore currently the fastest method for EM enumeration [85]. The mathematical rigor associated with the bit pattern tree method and other EMA algorithms has been described previously [86-88]. The code was acquired from Professor Jörg Stelling's website http://www.csb.ethz.ch/tools/efmtool and interfaced with The MathWorks™ MATLAB software (version 7.6.0.324).

### Pathway Gibbs Free Energy Calculations

The group contribution method of Mavrovouniotis was used in this study to estimate the standard Gibbs free energy of reaction for all of the model reactions [55]. Briefly, the group contribution method estimates the Δ*G*_*f*_° of metabolites by decomposing a single molecular structure into a subset of smaller functional groups, each individually contributing to overall Δ*G*_*f*_° values. The Δ*G*_*r*_° is then known as a result of the known stoichiometry of the reaction in question. Although currency metabolites were not included in the stoichiometric model, they were accounted for in the Gibbs free energy calculations to ensure consistency with reported data. All of the metabolites used in the stoichiometric models utilized here had corresponding Δ*G*_*f*_° values reported recently [56].

### Genetic Algorithm

Chromosomal representation of the metabolic genotype for passing to the genetic algorithm is binary in nature where a "1" indicates the reaction is included in the individual and "0" indicates that the reaction is not present. For simplicity's sake, a one-to-one association between reactions in the network and genes in the GA's population was assumed. This one-to-one association decreases computation time by utilizing fewer variables for optimization. This one-to-one association does not present a significant problem experimentally, for the gene-associations with the enzymes catalyzing the reactions are well-known for *E. coli *due to the organism's biochemical knowledge and sequenced genome [89-91]. A binary vector of length *n *therefore represents a single individual in the GA population.

Initialization of a population is a critical step for determining the success of the algorithm to find the global optimum. An initial population of fifty individuals containing between two and six knockouts was seeded to the algorithm (using MATLAB's "randerr" function). This was arrived at empirically as randomly seeding individuals with approximately 50% 0's resulted in mostly non-viable strains and did not allow for the GA to reach the optimal solution. Next, each individual in the population is evaluated and given a fitness score. A previous study on using GAs to optimize genotypic space for succinate, glycerol, and vanillin production used product flux determined by optimization (FBA and MoMA) as a scoring function [46]. As stated before, this approach relies on assumptions that may or may not be valid. Here, EMA was used as the method for scoring the individuals with fitness functions as described below.

Genetic algorithms use crossover of the chromosomes (mixing of two individuals in a population to create a new individual) and mutation (change a "0" to "1" and vice-versa with a specified frequency) to evolve the solution population. The implementation here was interfaced with The MathWorks™ MATLAB software and its Genetic Algorithm & Direct Search Toolbox. For crossover, mutation, and selection of individuals, two-point, uniform, and tournament-based methods were used, respectively. These parameters were not optimized in this study. As stated, the population size was chosen as fifty individuals, with five of the top performing individuals automatically passed to the next generation of the GA. The selection function used in the GA was either roulette- or tournament-based. The GA always terminated as a result of being below the tolerance (of the MATLAB default, 10^-6^) which was always between 50 and 100 generations.

As a method to reduce the computation time of the GA optimization, the GA was forced to always include (through fixed inclusion of a "1" in the individual genotype) reactions that were determined to either 1) reduce maximal product yield to zero, or 2) reduce maximal biomass yield to zero (indicating a lethal knockout). This reduced the genotypic space from 60 to 49 variables in the ethanol case study and 64 to 52 variables in the lycopene case study.

### Flux Determination & Fitness Function Selection

For the ethanol and lycopene case-studies, three fitness functions were examined utilizing both equal-weighting of the EMs as well as thermodynamically-weighted EMs. The flux vector can be recreated by taking the linear algebra inner-product of the EM matrix, *M*, with a weighting-vector, *c*. Here, *n *is the number of EMs.(1)

The differences in the two methods are in how the weighting vector, *c*, is determined. For the equal-weighting method:(2)

For the case in which the EMs are weighted by thermodynamic calculations, the Δ*G*_*p*_° values must be calculated from the Δ*G*_*r*_° values:(3)

Next, because it was previously determined that there existed a logarithmic relationship between the weighting factor of a particular EM and its contribution to the overall flux distribution with the change in entropy of the pathway, the weighting factor vector, *c*, is calculated with the following relationship:(4)

Here, the *T *represents for temperature, which was taken to be 310.15K (37°C, the optimal temperature for *E. coli *growth). To satisfy the constraint that the sum of the weighting vector must be equal to unity, the weighting vector is then divided by the sum of the weighting vectors.

Three different fitness functions with different goals were examined. The fitness functions corresponding to Eq. 5, Eq. 6, and Eq. 7 are all non-dimensionalized to unity by dividing by the theoretical product of biomass yields on substrate. However, achieving the theoretical yield of *both *a product *and *biomass on a particular substrate is impossible. Equation 5 was used to optimize a network structure for yield of product, *P*, on a substrate, *S *(in this case, glucose).(5)

Equation 6 was used to optimize for the biomass (*X*) yield on substrate:(6)

While both of the first two fitness functions are relevant for testing the functionality of the model here, a metabolic network that produces biomass and no product, or vice-versa, is not desirable. To optimize both biomass and product formation, a new fitness function was created as the product of Eqs. 5 and 6, which equally weights both biomass yield and product yield. In taking this step, the case where a cell contains a metabolic network incapable of producing either product or biomass is prevented:(7)

The computation time for these GA simulations were between 5-20 min on a notebook equipped with an Intel^® ^Core™ 2 Duo T9300 CPU running at 2.50 GHz, 4.0 GB memory, and a 32-bit version of Microsoft Windows Vista™ Ultimate.

### Flux Balance Analysis

Flux balance analysis is a linear programming method in which metabolic fluxes are determined by optimizing for biomass formation (maximizing growth rate) [92]. This was accomplished utilizing the "linprog" MATLAB function on the ethanol 47 × 60 stoichiometric matrix. For reversible reactions, a lower flux limit of -10 (arbitrary units) was used, while for irreversible reactions, a lower limit of 0 was used. For both reversible and irreversible reactions, the upper limit was chosen to be 10. The glucose uptake rate was fixed to 1 so as to scale the fluxes to glucose uptake rate and compare to the fluxes determined through weighting of the EMs. The general problem is posed as the following:(8)

Here, *S *is the stoichiometric matrix as described as previously and *v *is the flux vector.

Maximize: *z *= *c*^*T*^*v*(9)

In this optimization framework, *c *is a row vector containing weighting factors for individual fluxes on the objective function, *z*. For FBA calculations, this objective is solely the biomass reaction flux. *a*_*i *_and *b*_*i *_are the lower and upper bounds, respectively, of each flux as determined by either thermodynamics or experimental measurements.

### Multiobjective Genetic Algorithm

The MOGA invoked the same crossover, mutation, and selection algorithms as in the single-objective GA. Here, 200 individuals were used per population and the initial population was seeded randomly using between two and six removed reactions. The MOGA was run on the ethanol model using both equal-weighted and thermodynamic-weighted EMs subject to the two following fitness functions (those from Eq. 5 and Eq. 6):(10)(11)

The computation time for these MOGA simulations was much greater than the single-objective GA simulations, as expected. These simulations generally terminated after approximately 48 hours running on the same computer system described above.

## Abbreviations

Δ*G*_*f*_°: standard change in Gibbs free energy of formation; Δ*G*_*p*_°: standard change in Gibbs free energy across a pathway/EM; Δ*G*_*r*_°: standard change in Gibbs free energy across a reaction; Δ*S*_*p*_°: standard change in entropy across a pathway/EM; EM: elementary mode; EMA: elementary mode analysis; FBA: flux balance analysis; GA: genetic algorithm; MFA: metabolic flux analysis; MOGA: multi-objective genetic algorithm; MoMA: minimization of metabolic adjustment; NSGA-II: non-dominated sorting genetic algorithm-II; ROOM: regulatory on/off minimization; TRN: transcriptional regulatory network; TR-TR: transcriptional and translational.

## Authors' contributions

BAB conceived of the study, participated in its design, performed simulations, and helped draft the manuscript. HS calculated thermodynamic values. KL conceived of the study, participated in its design, and helped draft the manuscript. BAP participated in the study's design and helped draft the manuscript. All authors read and approved the final manuscript.

## Supplementary Material

Additional file 1 **Ethanol model information**. A table describing the ethanol model containing reaction number, abbreviation, stoichiometry, as well as corresponding gene names and enzymes. Click here for file

Additional file 2 **Lycopene model information**. A table describing the lycopene model containing reaction number, abbreviation, stoichiometry, as well as corresponding gene names and enzymes. Click here for file
