# Supplementary material for: Utilizing elementary mode analysis, pathway thermodynamics, and a genetic algorithm for metabolic flux determination and optimal metabolic network design
Source: BMC Syst Biol. 2010 Apr 23;4:49. doi: 10.1186/1752-0509-4-49 (PMC2880971; doi:10.1186/1752-0509-4-49)
Supplement: Additional file 2 — Lycopene model information. A table describing the lycopene model containing reaction number, abbreviation, stoichiometry, as well as corresponding gene names and enzymes. [file 1752-0509-4-49-S2.DOC]

*Table S2* (A list of the reaction abbreviations, stoichiometry, genes, and encoded enzymes in the lycopene model. *^a^*Biomass reaction stoichiometry is the same as described in *Table S1*. ^ext^ Indicates external metabolites which are not balanced in the analyses presented. ^het^ Indicates reactions that are catalyzed by heterologous enzymes.)

| **Number** | **Reaction Abbreviation** | **Reaction Stoichiometry** | **Gene(s)** | **Encoded Enzyme(s)** |
| --- | --- | --- | --- | --- |
| 1 | ANA1 | Phosphoenolpyruvate + CO_2_ → oxaloacetate | *ppc* | Phosphoenolpyruvate carboxylase |
| 2 | ANA2 | Malate + NAD^+^ → pyruvate + CO_2_ + NADH | *sfcA, maeB* | Malate dehydrogenase |
| 3 | ANA3 | Oxaloacetate + ATP → phosphoenolpyruvate + ADP + CO_2_ | *pckA* | Phosphoenolpyruvate carboxykinase |
| 4 | BIO | *a* |  | Biomass formation |
| 5 | EDP1 | 6-Phosphate gluconate → 2-keto-3-deoxy-6-phospho-gluconate | *edd* | Phosphogluconate dehydratase |
| 6 | EDP2 | 2-Keto-3-deoxy-6-phospho-gluconate → pyruvate + glyeraldehyde-3-phosphate | *eda* | 2-Keto-3-deoxy-6-phosphogluconate aldolase |
| 7 | FC1r | NAD^+^ + NADPH ↔ NADP^+^ + NADH | *pntAB* | Pyridine nucleotide transhydrogenase |
| 8 | FC2 | AMP + ATP → 2 ATP | *adk* | Adenylate kinase |
| 9 | FEM1 | Pyruvate + CoASH → acetyl-CoA + CO_2_ + quinol | *pflB, tdcE* | Pyruvate formate-lyase |
| 10 | FEM2 | Pyruvate + quinine → acetate + CO_2_ + quinol | *poxB* | Pyruvate oxidase |
| 11 | FEM3 | Pyruvate + NADH → lactate + NAD^+^ | *ldhA* | Lactate dehydrogenase |
| 12 | FEM4 | Formate + CO_2_ → H_2_^ext^ | *hycBCDEFG*  *fdhF* | Formate dehydrogenase |
| 13 | FEM5 | Acetyl-CoA + NADH → acetaldehyde + NAD^+^ +CoASH | *adhE* | Acetaldehyde dehydrogenase |
| 14 | FEM6 | Acetaldehyde + NADH → ethanol + NAD^+^ | *adhEP* | Alcohol dehydrogenase,  ethanol dehydrogenase |
| 15 | FEM7 | Acetyl-CoA → acetylphosphate + CoASH | *pta* | Phosphate acetyltransferase |
| 16 | FEM8 | Acetylphosphate + ADP → acetate + ATP | *ackAB* | Acetate kinase |
| 17 | GG1 | Glucose^ext^ + phosphoenolpyruvate → glucose-6-phosphate + pyruvate | *ptsGHI, crr* | Phosphoenolpyruvate:glucose transferase system |
| 18 | GG2r | Glucose-6-phosphate ↔ fructose-6-phosphate | *pgi* | Phosphoglucose isomerase |
| 19 | GG3 | Fructose-6-phosphate + ATP → fructose-1,6-bisphosphate | *pfkAB* | 6-Phosphofructokinase |
| 20 | GG4 | Fructose-1,6-bisphosphate → fructose-6-phosphate | *glpX, fbp* | Fructose-1,6-biphosphatase |
| 21 | GG5r | Fructose-1,6-bisphosphate ↔ dihydroxy-acetone-phosphate + glyeraldehyde-3-phosphate | *fbaAB* | Fructose bisphosphate aldolase |
| 22 | GG6r | Glyeraldehyde-3-phosphate ↔ dihydroxy-acetone-phosphate | *tpiA* | Triose phosphate isomerase |
| 23 | GG7r | Glyeraldehyde-3-phosphate + NAD^+^ ↔ 3-phospho-glyceroyl-phosphate + NADH | *gapA* | Glyceraldehyde 3-phosphate dehydrogenase |
| 24 | GG8r | 3-Phospho-glyceroyl-phosphate + ADP ↔ 3-phosphoglycerate | *pgk* | Phosphoglycerate kinase |
| 25 | GG9r | 3-phosphoglycerate ↔ 2-phosphoglyerate | *pgml, ytjC, pgmA* | Phosphoglycerate mutase |
| 26 | GG10r | 2-Phosphoglyerate ↔ phosphoenolpyruvate | *eno* | Enolase |
| 27 | GG11 | Phosphoenolpyruvate + ADP → pyruvate + ATP | *pykAF* | Pyruvate kinase |
| 28 | GG12 | Pyruvate + ATP → Phosphoenolpyruvate + AMP | *pps* | Phosphoenolpyruvate synthase |
| 29 | GG13 | Pyruvate + CoASH + NAD^+^ → acetyl-CoA + CO_2_ + NADH | *lpdA, aceEF* | Pyruvate dehydrogenase complex |
| 30 | GLB1 | Isocitrate → glyoxylate + succinate | *aceA* | Isocitrate lyase |
| 31 | GLB2 | Glyoxylate + acetyl-CoA → malate + CoASH | *glcB, aceB* | Malate synthase |
| 32 | OPM1 | NADH + 2 ADP + O_2_^ext^ → NAD^+^ + 2 ATP | *nuoAHJKLMNEFGBCI,*  *atpABCDEFGHI* | NADH dehydrogenase I  ATP synthase |
| 33 | OPM2 | Quinol + ADP + O_2_^ext^ → quinine + ATP | *cyoABCD, atpABCDEFGHI* | Cytochrome bo terminal oxidase  ATP synthase |
| 34 | OPM3 | ATP → ADP + ATP^main^ |  | Maintenance energy |
| 35 | OPM4r | NADH + quinine ↔ NAD NAD^+^ + quinol | *ndh* | NADH dehydrogenase II |
| 36 | PPP1 | Glucose-6-phosphate + NADP^+^ → glucono-lactone-6-phosphate + NADPH | *zwf* | Glucose 6-phosphate-1-dehydrogenase |
| 37 | PPP2 | Glucono-lactone-6-phosphate → 6-phosphate gluconate | *pgl* | 6-Phosphoglucolactonase |
| 38 | PPP3 | 6-Phosphate gluconate + NADP^+^ → ribulose-5-phosphate + CO_2_ + NADPH | *gnd* | 6-Phosphogluconate dehydrogenase |
| 39 | PPP4r | Ribulose-5-phosphate ↔ xylulose-5-phosphate | *rpe* | Ribulose phosphate 3-epimerase |
| 40 | PPP5r | Ribulose-5-phosphate ↔ ribose-5-phosphate | *rpiA, alsI* | Ribose-5-phosphate isomerase |
| 41 | PPP6r | Ribose-5-phosphate + xylulose-5-phosphate ↔ sedoheptulose-7-phosphate + glyeraldehyde-3-phosphate | *tktAB* | Transketolase |
| 42 | PPP7r | Glyeraldehyde-3-phosphate + sedoheptulose-7-phosphate ↔ erythrose-4-phosphate + fructose-6-phosphate | *talAB* | Transadolase |
| 43 | PPP8r | Erythrose-4-phosphate + xylulose-5-phosphate ↔ glyeraldehyde-3-phosphate + fructose-6-phosphate | *tktAB* | Transketolase |
| 44 | TCA1 | Oxaloacetate + acetyl-CoA → citrate + CoASH | *prpC, gltA* | Methylcitrate synthase,  Citrate synthase |
| 45 | TCA2r | Citrate ↔ *cis*-aconitate | *acnAB* | Aconitase |
| 46 | TCA3r | *cis*-Aconitate ↔ isocitrate | *acnAB* | Aconitase |
| 47 | TCA4 | Isocitrate + NADP^+^ → α-ketoglutarate + CO_2_ + NADPH | *icd* | Isocitrate dehydrogenase |
| 48 | TCA5 | α-Ketoglutarate + NAD^+^ + CoASH → NADH + succinyl-CoA + CO_2_ | *lpdA, sucAB* | α-Ketoglutarate dehydrogenase complex |
| 49 | TCA6r | Succinyl-CoA + ADP ↔ succinate + ATP + CoASH | *sucCD* | Succinyl-CoA synthase |
| 50 | TCA7 | Succinate + quinine → fumarate + quinol | *sdhABCD* | Succinate dehydrogenase |
| 51 | TCA8r | Fumarate ↔ malate | *fumABC* | Fumarase A |
| 52 | TCA9r | Malate + NAD^+^ ↔ oxaloacetate + NADH | *mdh* | Malate dehydrogenase |
| 53 | TCA10 | Fumarate + quinol → succinate + quinine | *frdABCD* | Fumarate reductase |
| 54 | TRA1 | Ethanol → ethanol^ext^ |  | Ethanol transport |
| 55 | TRA2 | Acetate → acetate^ext^ |  | Acetate transport |
| 56 | TRA3 | NH_3_^ext^ → NH_3_ |  | Ammonia transport |
| 57 | TRA4 | Lactate → lactate^ext^ |  | Lactate transport |
| 58 | TRA5 | Succinate → succinate^ext^ |  | Succinate transport |
| 59 | TRA6 | Formate → formate^ext^ |  | Formate transport |
| 60 | TRA7 | CO_2_ → CO_2_^ext^ |  | Carbon dioxide transport |
| 61 | LYCO1 | Glyceraldehyde-3-phosphate + pyruvate + 2 NADPH + ATP → dimethylallyl diphosphate + CO_2_ + 2 NADP^+^ + ADP | *dxs, ispCDEFGH* | Non-mevalonate isoprenoid pathways |
| 62 | LYCO2r | Dimethylallyl diphosphate ↔ isopentyl diphosphate | *idi* | Isopentenyl diphosphate isomerase |
| 63 | LYCO3^het^ | 4 Isopentyl diphosphate → geranylgeranyl diphosphate | *ispA, crtE* | Geranyl diphosphate synthase,  geranylgeranyl diphosphate synthase |
| 64 | LYCO4^het^ | 2 geranylgeranyl diphosphate + 8 NADPH → lycopene^ext^ + 8 NADP^+^ | *crtBI* | Phytoene synthase,  phytoene dehydrogenase |
